# Association of *NLRP1* Coding Polymorphism with Lung Function and Serum IL-1β Concentration in Patients Diagnosed with Chronic Obstructive Pulmonary Disease (COPD)

**DOI:** 10.3390/genes10100783

**Published:** 2019-10-09

**Authors:** Petar Ozretić, Miguel Inacio da Silva Filho, Calogerina Catalano, Irena Sokolović, Andrea Vukić-Dugac, Maja Šutić, Matea Kurtović, Gordana Bubanović, Sanja Popović-Grle, Sanda Skrinjarić-Cincar, Oliver Vugrek, Irena Jukić, Lada Rumora, Martina Bosnar, Miroslav Samaržija, Robert Bals, Marko Jakopović, Asta Försti, Jelena Knežević

**Affiliations:** 1 Ruđer Bošković Institute, Division of Molecular Medicine, 10 000 Zagreb, Croatia; Petar.Ozretic@irb.hr (P.O.); Maja.Sutic@irb.hr (M.Š.); Matea.Kurtovic@irb.hr (M.K.); Oliver.Vugrek@irb.hr (O.V.); 2 Division of Molecular Genetic Epidemiology, DKFZ, 69 120 Heidelberg, Germany; miguelisifi@yahoo.com (M.I.d.S.F.); c.catalano@dkfz-heidelberg.de (C.C.); 3 Department for Respiratory Diseases Jordanovac, University of Zagreb School of Medicine, University Hospital Centre Zagreb, 10 000 Zagreb, Croatia; isokolovic2@gmail.com (I.S.); adugac71@gmail.com (A.V.-D.); gordana.bubanovic@kbc-zagreb.hr (G.B.); sanja.popovic.grle@kbc-zagreb.hr (S.P.-G.); miroslav.samarzija@gmail.com (M.S.); marko.jakopovic@kbc-zagreb.hr (M.J.); 4 Josip Juraj Strossmayer University of Osijek, School of Medicine, 31 000 Osijek, Croatia; sanda.cincar@gmail.com; 5 Department of Pulmology, Universitiy Hospital Center Osijek, 31 000 Osijek, Croatia; 6 Croatian Institute of Transfusion Medicine, 10 000 Zagreb, Croatia; irena.jukic@hztm.hr; 7 Department of Medical Biochemistry and Hematology, Faculty of Pharmacy and Biochemistry, University of Zagreb, 10 000 Zagreb, Croatia; ladarumora@gmail.com; 8 Fidelta d.o.o., Prilaz baruna Filipovića 29, 10 000 Zagreb, Croatia; Martina.Bosnar@glpg.com; 9 Department of Internal Medicine V-Pulmonology, Allergology, Intensive Care Medicine, Saarland University, 66 424 Homburg, Germany; Robert.Bals@uks.eu

**Keywords:** COPD, *NLRP*, polymorphism, FEV_1_, FEV_1_/FVC, GOLD, serum IL-1β

## Abstract

Chronic obstructive pulmonary disease (COPD) is a chronic disease characterized by a progressive decline in lung function due to airflow limitation, mainly related to IL-1β-induced inflammation. We have hypothesized that single nucleotide polymorphisms (SNPs) in *NLRP* genes, coding for key regulators of IL-1β, are associated with pathogenesis and clinical phenotypes of COPD. We recruited 704 COPD individuals and 1238 healthy controls for this study. Twenty non-synonymous SNPs in 10 different *NLRP* genes were genotyped. Genetic associations were estimated using logistic regression, adjusting for age, gender, and smoking history. The impact of genotypes on patients’ overall survival was analyzed with the Kaplan–Meier method with the log-rank test. Serum IL-1β concentration was determined by high sensitivity assay and expression analysis was done by RT-PCR. Decreased lung function, measured by a forced expiratory volume in 1 s (FEV_1_% predicted), was significantly associated with the minor allele genotypes (AT + TT) of *NLRP1* rs12150220 (*p* = 0.0002). The same rs12150220 genotypes exhibited a higher level of serum IL-1β compared to the AA genotype (*p* = 0.027) in COPD patients. *NLRP8* rs306481 minor allele genotypes (AG + AA) were more common in the Global Initiative for Chronic Obstructive Lung Disease (GOLD) definition of group A (*p* = 0.0083). Polymorphisms in *NLRP1* (rs12150220; OR = 0.55, *p* = 0.03) and *NLRP4* (rs12462372; OR = 0.36, *p* = 0.03) were only nominally associated with COPD risk. In conclusion, coding polymorphisms in *NLRP1* rs12150220 show an association with COPD disease severity, indicating that the fine-tuning of the NLRP1 inflammasome could be important in maintaining lung tissue integrity and treating the chronic inflammation of airways.

## 1. Introduction

Chronic obstructive pulmonary disease (COPD) is currently a leading cause of death [1]. The World Health Organization (WHO) predicts that by the year 2030 COPD will be the third leading cause of death in the world [2]. COPD is a chronic inflammatory disease leading to structural abnormalities in the airways, and it is associated with exposure to noxious particles, like cigarette smoke [3]. The disease is characterized by a progressive and irreversible decline in lung function due to airflow obstruction and parenchyma destruction [4]. The genetic basis of innate immune response is now a well-established contributor in disease pathogenesis, since only 15–20% of smokers develop COPD, and chronic inflammation persists even after smoking cessation [5]. COPD is associated with different comorbidities that affect the clinical phenotype of the disease, its course, and outcome [6]. The causal relationship between comorbidities and COPD remains unclear [7]. It is known that the clinical course, prognosis, and overall survival of COPD patients are all influenced by comorbidities, many of which are accompanied by systemic inflammation [8]. The respiratory innate immune cells constantly respond to a broad spectra of inhaled microbes and air pollutants. They sense potential threats or ‘dangers’ through the expression of the receptors known as pattern-recognition receptors (PRRs). PRRs play a role in the recognition of pathogen-associated molecular patterns (PAMPs) and damage-associated molecular patterns (DAMPs) [9]. Upon the recognition of PAMPs or DAMPs, PRRs elicit the activation of the innate immune response to eliminate the source of danger and restore tissue homeostasis. Deficient mucosal immunity can cause serious infections, while the persistent exposure of the respiratory epithelium to environmental factors contributes to pulmonary diseases, like COPD. Nod-like receptors (NLRs) are PRRs that can recognize both PAMPs and DAMPs. NLRPs, which are members of the NLR family, comprise a group of 14 proteins, contributing to both the inflammatory and tissue remodeling processes [10]. NLRPs can be activated by a broad range of stimuli, from bacterial products to a number of stress- and damage-associated signals, culminating in inflammasome formation and the caspase-1 proteolytic cleavage of the IL-1β precursor [11]. Generally, five main inflammasomes are identified: NLRP1, NLRC4, RIG-1, AIM2, and NLRP3. When activated, upon exposure to structurally different PAMPs or DAMPs, they act as scaffolds to form active protein complexes together with an adaptor protein, namely, an apoptosis-associated speck-like protein containing a caspase recruitment domain (ASC), and pro-caspase-1 [12]. In different COPD models, many studies have demonstrated that IL-1β significantly contributes to airway inflammation and emphysema, where both stabile and exacerbating COPD IL-1β secretion is increased [13]. Also, IL-1β induces the secretion of IL-8 and IL-6, two cytokines that promote neutrophil recruitment, a process that significantly contributes to neutrophilic airway inflammation [14]. However, the relative contribution of different inflammasomes in inducing IL-1β responses and their roles in pathogenesis of COPD are still elusive [15]. Hence, it is conceivable that the genetic variability of the key regulators of IL-1β, i.e., NLRPs, could be associated with COPD and its different clinical phenotypes. The aim of this study was to perform a genetic association study on the basis of COPD in a case-control cohort and its disease conditions in the case-only cohort, in the context of the single nucleotide polymorphism (SNP) frequency of the *NLRP* genes. The genetic background was examined in the context of the single nucleotide polymorphism (SNP) frequency of the *NLRP* genes.

## 2. Materials and Methods

### 2.1. Study Population

The demographic characteristics of the study population are shown in Table 1 and the clinical characteristics of the COPD patients are shown in Table 2. The diseased population was recruited by the Department for Respiratory Diseases, in the Clinical Hospital Centre in Zagreb, and the Department for Pulmonology, Clinical Hospital Centre Osijek, Croatia. Genotyping was conducted on 527 COPD cases, together with 1238 healthy controls, which were collected at the Department of Transfusion Medicine, Zagreb, Croatia. COPD diagnosis and its stage were defined according to the Global Initiative for Chronic Obstructive Lung Disease (GOLD) criteria (Update 2017). Spirometry was performed according to the American Thoracic Society/European Respiratory Society (ATS/ERS) criteria. A post-bronchodilator FEV1/FVC ratio less than 70 was considered as a diagnosis of airflow limitation. Phenotype evaluation was done by pulmonary function tests, with the clinical data obtained for three cardinal symptoms (dyspnea, chronic cough and sputum production), the annual exacerbation rate, and performance status. Patient assessment included their past medical history, covering significant comorbidities, exposure to risk factors, physical examination, and smoking status. Age at onset was also registered. The follow-up data used for overall survival (OS) were obtained from medical records. Survival data were obtained for 525 patients, of whom 81 died during the follow-up period. The median follow-up time of the patients was 81 months (range 1–451 months). OS time was measured from the date of diagnosis to the time of death by any cause. Comorbidity was defined as the presence of one or more distinct disorders or diseases in addition to COPD. The control group of healthy volunteers, recruited during the regular blood donation process by the Department of Transfusion Medicine, Zagreb, represents the general healthy population characterized by good basic health status. Patients recruited for serum and RNA isolation needed to meet an additional inclusion criterion. For the COPD cohort (N = 100), this criterion was the stable state of the disease, defined as having no symptoms that could be correlated with exacerbation over at least 4–6 weeks, while for the healthy control subjects (N = 100), the exclusion criterion was a history of acute pulmonary infection or any other infection in the last 6 weeks before assessment. This study was performed in accordance with the Declaration of Helsinki. The study was approved by the ethical committees of University Hospital Centers Zagreb and Osijek and Croatian Institute of Transfusion Medicine. All participants provided written informed consent to participate in this study.

### 2.2. Gene/SNP Selection and Genotyping

**Figure 1:**
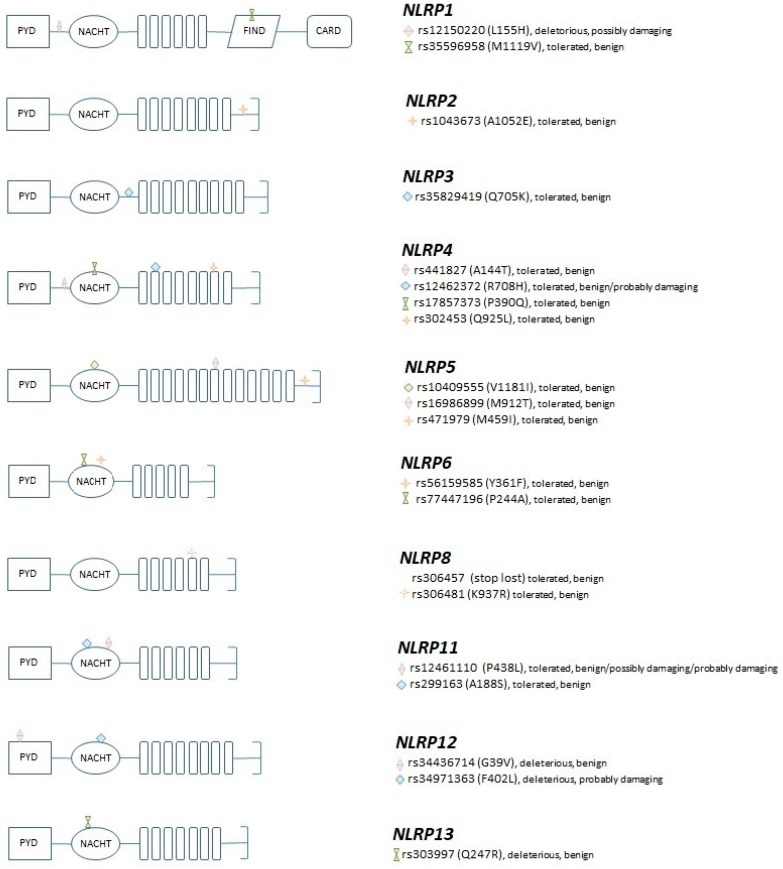
The structure of the selected *NLRP* genes, the localization of the genotyped SNPs, and their predicted functional consequences according to SIFT and PolyPhen, respectively.

The gene selection criterion was based on the hypothesis that the genetic diversity of NLRPs is implicated in the dysregulated activation/regulation of respiratory inflammation. Twenty SNPs located in 10 *NLRP* genes were tested (Table 3). All tested SNPs are missense variants, and they are located in the coding regions, with a minor allele frequency higher than 1% (based on the dbSNP database (NCBI, Bethesda, MD, USA) (http://www.ncbi.nlm.nih.gov/snp), and only 1 SNP per linkage block was selected for genotyping. Out of the 20 selected SNPs, 15 were located in the specified NLRP domains (1 in FIIND, 5 in LRR and 9 in NACHT domain), while the others were located in the linker domains of the proteins (Figure 1). More details on the selection criteria can be found in [16]. The deleteriousness of the amino acid changes were predicted using SIFT (http://sift.jcvi.org/) and PolyPhen-2 (http://genetics.bwh.harvard.edu/pph2/). Genotyping was performed using the previously published protocol [17].

### 2.3. Gene Expression Analyses

The gene expression analyses were performed on RNA samples isolated from peripheral blood mononuclear cells (PBMCs) from COPD patients and healthy donors, as well as from the human lung fibroblast cell line (WI38?; obtained from ATCC) using TaqMan Gene expression assays (Thermo Fisher) for the *NLRP1*, *NLRP4*, and *NLRP8* genes. The WI38 cell line was selected for the study because fibroblasts are believed to be the major cells responsible for the production and maintenance of the extracellular matrix, and fibroblasts from individuals with COPD have a reduced capability to sustain tissue repair [18]. The total RNA, from blood, was extracted using Trizol. A RNeasy Mini Kit (Qiagen) was used for RNA extraction from human lung fibroblast WI38. Reverse transcription was done with a High Capacity cDNA Reverse Transcription Kit (Applied Biosystems). The analyses were performed in triplicate using the 7300 Real-time PCR System (Applied Biosystems).

### 2.4. Serum IL-1β Concentration

Concentrations of IL-1β in the sera of COPD patients and healthy donors were measured using a ProcartaPlex High Sensitivity Assay, with a corresponding IL-1b bead set (Thermo Fisher Scientific, Waltman, MA, USA), according to manufacturer’s recommendation. Briefly, 50 µL of antibody-coated magnetic beads were added per well into a 96-well plate and washed. Afterwards, 25 µL of samples or standards were added to a 25 µL universal assay buffer, and the plate was incubated for 30 min at room temperature (RT) and overnight at 4 °C, with shaking. After the washing steps, 25 µL of detection antibodies were added to the wells and the plate was incubated for 30 min at RT, with shaking. After the washing, 50 µL of a streptavidin-phycoerythrin conjugate was added to the wells. After the incubation and washing steps, 50 µL of amplification reagent 1 was added to the wells, and the plate was incubated for 30 min at RT, with shaking. Then, amplification reagent 2 (50 µL) was added to the wells, and following the incubation and washing steps, the beads were resuspended in a 120 µL reading buffer and analyzed by use of a Luminex 200 instrument. The concentration of IL-1β was determined by interpolation from a standard curve using the xPONENT software package (Luminex, Austin, TX, USA).

### 2.5. Statistical Analysis

Associations between the genotyped markers and disease were estimated as odds ratios (ORs) with 95% confidence intervals (CIs) using logistic regression. These were adjusted for gender, age, and smoking history. The associations were calculated for dominant and codominant models. The χ^2^ test was used to assess the distribution and association of categorical variables. Since the continuous variables did not show a normal distribution, which was tested with the D’Agostino-Pearson test, the non-parametric Mann–Whitney test, and the non-parametric Kruskall–Wallis test, with a post-hoc test according to Conover, which was used to determine if there were statistically significant differences among the genotypes. The median was used as a cut-off value to dichotomize continuous variables. *p* values below 0.0025 were considered statistically significant (Bonferroni correction), while the nominal association was assigned to *p* values below 0.05. For assessing the influence of genotypes on patients’ overall survival, the Kaplan–Meier method was used for calculating survival curves, which were compared by the log-rank test. Two-tailed *p*-values <0.05 were considered statistically significant, while for the pairwise comparison of GOLD status, the Bonferroni correction was applied. The statistical analyses were performed using MedCalc version 18.2.1 (MedCalc Software, Ostend, Belgium) and SAS, software version 9.2 (SAS Institute, Heidelberg, Germany).

## 3. Results

### 3.1. Association of the NLRP Coding Variants with COPD Risk and Disease Severity

**Figure 2:**
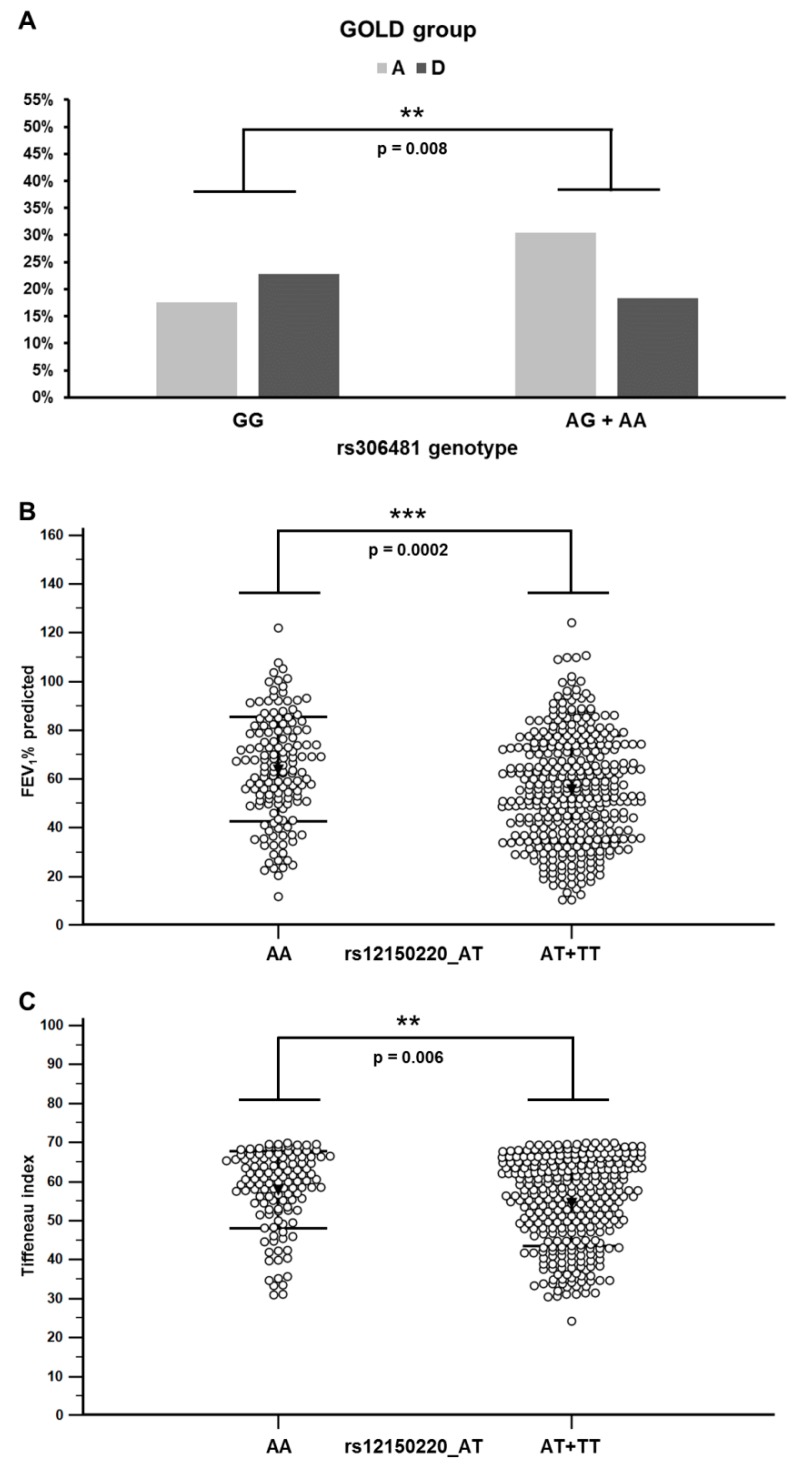
Distribution of the GOLD groups among rs306481 genotypes and the association of the rs12150220 genotypes with spirometric parameters in COPD patients. (**A**) Allele A carrier status in the tested locus correlated with the milder GOLD stages; refer to Table 3 for the results of the statistical analysis. Numbers present the percentage of individuals with indicated GOLD status. (**B**) Patients with genotypes involving minor allele T showed lower FEV_1_ predicted values. (**C**) Patients with genotypes involving minor allele T showed lower FEV_1_/FVC ratio (Tiffeneau index). Bars represent standard deviation and the black triangle represents the mean value. Stars indicate statistically significant differences (*p* < 0.05).

We genotyped 20 SNPs located in 10 different NLRP genes (Table 1). Logistic regression analysis, adjusted for age, gender, and smoking history, indicated only a nominal association with COPD risk for two SNPs in dominant model: NLRP1 SNP rs12150220_AT (A/T OR = 0.54, *p* = 0.03; T/T OR = 0.59, *p* = 0.17; A/T + T/T OR = 0.55, *p* = 0.03) and NLRP4 SNP rs12462372_AG (A/G OR = 0.36, *p* = 0.03; A/A OR = 0.29, *p* = 0.77; A/G + A/A OR = 0.36, *p* = 0.03), suggesting that there is no association between the tested SNPs and COPD risk (Table 4). However, when we performed an association study of the NLRP SNPs and disease conditions that are important predictors of COPD severity and disease outcome, such as the GOLD groups (ABCD groups), the predicted FEV_1_%, and FEV_1_/FVC ratio (Tiffeneau index), we found that the SNPs located in the NLRP1 and NLRP8 genes were associated with GOLD, FEV_1_%, and the FEV_1_/FVC ratio. We found a significant difference between the frequencies of the genotypes of NLRP8 polymorphism rs306481 (*p* = 0.0002; chi-square test) in the GOLD groups (ABCD), while NLRP1 rs12150220 association was of borderline significance (*p* = 0.049) (Table S1). Pairwise analysis showed that the most significant difference in the distribution of the genotypes was for the NLRP8 SNP rs306481 between GOLD groups A and D (*p* = 0.0001). The subgroup analysis showed that genotypes harboring allele A (AG+AA) were more frequent among patients in GOLD group A, while genotype GG (*p* = 0.0083) was more frequent among patients in GOLD group D (Figure 2A). This suggests that the minor allele A may be protective, and that the major allele G is a risk allele for a more severe disease. We also detected that the FEV_1_% predicted values were differently distributed between the NLRP1 rs12150220 genotypes (*p* = 0.00014) and the post-hoc analysis, showing that genotypes harboring minor allele T (AT+TT) were associated with a lower FEV_1_% (*p* = 0.0002) (Figure 2B). Finally, we detected that FEV_1_/FVC ratio was differently distributed between the genotypes of the same polymorphism (rs12150220) (*p* = 0.0083). The post-hoc analysis (Mann–Whitney test) showed a significant association between the homozygous genotype rs12150220_AA (major allele) with higher values of the FEV_1_/FVC ratio, compared to genotypes harboring minor allele (AT+TT) (*p* = 0.0083) (Figure 2C). We did not find any association between the NLRP polymorphisms and overall survival (Supplementary Figure S1).

### 3.2. NLRP1 is Expressed in Human PBMCs and Lung Fibroblasts

**Figure 3:**
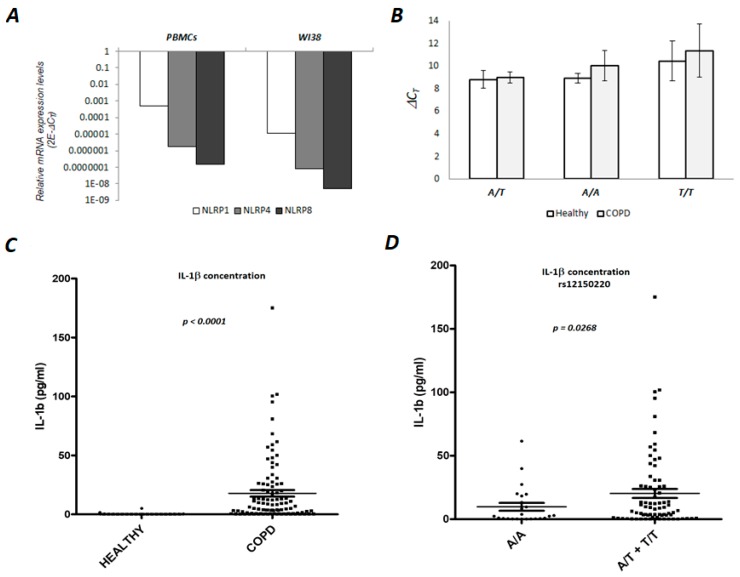
Expression analyses of selected genes in human PBMCs and lung fibroblast cells and serum IL-1β concentration. (**A**) Real-time PCR analysis of NLRP1, NLRP4 and NLRP8 mRNA was performed on cDNA samples from COPD patients (N = 4) and WI38 cell line (human lung fibroblasts). Total RNA was isolated either from peripheral blood mononuclear cells (PBMC) or cell line, and reverse-transcribed to cDNA. GAPDH was used as a reference or endogenous control. For quantification see Materials and Methods. (**B**) Real-time PCR analysis of NLRP1 mRNA expression levels. The expression analysis was performed on 15 cDNA samples from heathy individuals and equal number of COPD patients (RNA was isolated from PBMC). GAPDH was included as a reference or endogenous control. ∆C_T_ values of NLRP1 were normalized to GAPDH C_T_ values and ∆C_T_ is shown on the graph. Each column represents the average value of ∆C_T_ of 5 individuals with the given genotype (A/A, A/T or T/T). The experiment was conducted two times in duplicates. (**C**) Concentration of the serum IL-1β in healthy and COPD individuals. IL-1β concentration was determined in the plasma samples separated from the healthy donors (N76) and the individuals with diagnosed COPD (N = 100) (*NLRP1* genotype (rs12150220) was known for each sample). Analysis was performed with a ProcartaPlex High Sensitivity Assay, according to manufactures protocol, which can be found in the Materials and Methods section. (**D**) The concentration of serum IL-1β is related to rs12150220. The relationship between IL-1β concentration and *NLRP1* (rs12150220) genotypes AA (N = 24) vs. AT+TT (N = 76) in the COPD serum samples. AT and TT genotypes carriers had higher IL-1β levels compared to the AA genotype carriers.

We quantified mRNA levels in the immune cells separated from peripheral blood (PBMCs) from 4 COPD patients and the human lung fibroblast cell line (WI38). ∆C_T_ values (normalized to GAPDH C_T_ values), which should be reverse-proportional to mRNA expression, indicated that the PBMCs and the WI38 cell line express the *NLRP1* gene. The detected C_T_ values were 28–36 cycles (mean C_T_ = 30 cycles), indicating moderate expression. In the case of *NLRP4*, only a minimal expression level was observed, while in case of *NLRP8*, we did not detect any expression in any of the tested samples (C_T_ values were detected only above 40 cycles) (Figure 3A). In order to see if specific genotypes could be associated with a different expression level of *NLRP1* in PBMCs, 15 cDNA samples from COPD and equal number of healthy donors were analyzed. The results of this analysis indicated that there were no differences in the expression levels of *NLRP1* between healthy and COPD samples, and also that the expression was not influenced by different rs12150220 genotypes (Figure 3B). 

### 3.3. Serum Concentration of the IL-1β in the COPD Patients is Associated with NLRP1 rs12150220 Genotype

The proinflammatory cytokine interleukin IL-1β is the key mediator of neutrophilic airway inflammation in COPD. As NLRPs are key regulators of IL-1β, we wanted to examine if the serum level of IL-1β could be associated with NLRP1 (rs12150220) genotypes. First, we confirmed that serum IL-1β is significantly elevated in COPD patients in comparison to healthy donors (nonparametric Mann–Whitney test; *p* < 0.0001, Figure 3C). Due to the almost undetectable level of the serum IL-1β in healthy donors, they were excluded from further analyses. Next, we compared the frequency of minor allele genotypes (AT+TT) and the major allele (AA) genotype with serum IL-1β and found that major allele homozygosity was associated with lower IL-1β concentration (nonparametric t-Test; *p* = 0.03, Figure 3D). In conclusion, it seems that the presence of the minor allele could be associated with, to some extent, elevated levels of serum IL-1β. 

## 4. Discussion

The case-control and case-only association studies identified three coding SNPs, so far not analyzed in this context, that were associated with COPD disease severity and IL-1β concentration in the serum: *NLRP1* (rs12150220, L155H), *NLRP4* (rs12462372, R708H) and *NLRP8* (rs306481, K937R). Expression analysis showed that only the *NLRP1* gene exhibits moderate expression levels in both the PBMCs of COPD patients and the WI38 cell line. Therefore, the functional significance of the *NLRP4* and *NLRP8* polymorphisms in our case is not clear. In vitro studies, performed by others, have shown that NLRP4 is a negative regulator of type I interferon activity [19] and autophagy during A streptococcal infection [20]. However, it is hard to predict any functional consequence of NLRP4 in the lung, due to the fact that rs12462372 was also predicted to be tolerated and benign by SIFT and PolyPhen (Table 2). The same was true for NLRP8 rs306481, which we found to correlate with the GOLD groups. The functional consequences of this finding are difficult to comment on, since there is a lack of published data on NLRP8 function, probably due to the fact that its expression is lost in rodents [21]. Therefore, our report, based on a genetic association study, that the minor allele A could be considered as protective, should be interpreted with caution.

For us, the most interesting finding of this study was that *NLRP1* rs12150220 exhibited a statistically significant association with lung function, where minor allele carriers had a decreased FEV_1_% and FEV_1_/FVC ratio, together with, to some extent, elevated serum IL-1β-clinical conditions, which have been strongly correlated with worse COPD prognosis [22]. The fact that we did not find any association between the genotypes and overall survival does not contradict our findings regarding lung function and IL-1β levels, because the effects of individual SNPs are usually small compared to comorbidities or other strong prognostic markers. On the other hand, our results may indicate that the *NLRP1* SNP rs12150220 may, indirectly, affect survival by affecting COPD phenotypes. It is worth mentioning that more than 54% of COPD patients recruited in this study have more than two comorbidities, while more that 28% have one. The presence of different comorbidities significantly impacts patients’ quality of life, exacerbation frequency, survival, and, in general, clinical outcomes [23]. Many comorbidities that result from the chronic inflammatory state are present in COPD, and it is possible that persistent low-grade systemic inflammation may be the link between COPD and comorbidities [24]. However, the precise mechanisms of these processes are yet to be defined. It has been shown that obesity, which is found to be more frequent in the COPD group, affects patients’ clinical manifestations and quality of life, possibly by contributing to the phenotype group characterized by increased systemic inflammation [25].

The innate immune response in the lungs, orchestrated by NLRPs, is the key step in sensing infection, tissue damage, and different types of tissue integrity disturbance [26]. Defects in the NLRP1 inflammasome pathway have been linked to different pulmonary diseased conditions. Li et al. has shown that the activation of the NLRP1 and NLRP3 inflammasome pathways may contribute to pulmonary fibrosis caused by latent MCMV infection in mice [27]. Crovella et al. have investigated how the genetic variability of inflammasome encoding genes impacts the formation of asbestos bodies (Abs) in lung parenchyma, showing that the *NLRP1*rs12150220 missense variant significantly correlates with the number of ABs in malignant pleural mesothelioma patients [28]. Leal et al. found that the NLRP1 gain-of-function variants rs11651270, rs12150220, and rs2670660 are significantly associated to asthma, while rs11651270 and rs2670660 are associated with asthma severity and the total IgE level in asthmatic children [29]. Interestingly, it has also been shown that different autoimmune diseases, like vitiligo and systematic lupus erythematosus (SLE) are associated with defects in NLRP1 [30]. L155H (rs12150220) is located in the linker region, between PYD and NACHT domains of the NLRP1 receptor, and was predicted to be deleterious, as reported by Levandowski et al. [31]. They have shown that L155H mutation is associated with autoimmunity and increased IL-1β processing, which also agrees with our results. Cultured L155H/L155H peripheral blood monocytes, in unstimulated conditions, processed significantly greater amount of the pro-IL-1β to mature IL-1β, compared to wild type. The precise mechanism by which L155H affects inflammasome activation is not known. It is very likely that L155H haplotype is associated with the increased auto-proteolytic activation of L155H-NLRP1, resulting in the systemic activation of caspase-1 and increased IL-1β processing. However, beside IL-1β maturation, NLRP1 participates in the regulation of cell death. Zhai et al. have shown that the knocking down of NLRP1 in human melanoma cell lines promotes apoptosis [32], suggesting that enhanced NLRP1 inflammasome activity could be associated with the tumor-promoting role of NLRP1 in cancer cells. In addition, Kovarova et al. has shown that NLRP1-dependent pyroptosis leads to acute lung injury [33]. They showed that the lungs of Casp1^−/−^ mice were protected from developing acute respiratory distress, a catastrophic consequence of the NLRP1 activation, suggesting that caspase-1 activation, not only IL-1β processing, is also the critical outcome of NLRP1 activation. In the presented study, we have shown that *NLRP1* SNP rs12150220 exhibits only a nominal association with COPD risk, demonstrated with a *p* value of 0.03, which is not significant enough to pass the Bonferroni correction process. However, odds ratio values of 0.55 suggest a potential protective effect of the tested SNP in the COPD population. It remains to be further examined how *NLRP1* genotypes could be associated with a worse prognosis of COPD, as found in our study, and potential protective properties in COPD development. It is possible that the presence of a mutated allele could be generally protective, but in the case of ‘double-hits’, COPD and the mutated allele could be potentially detrimental, as was shown in our study. Nevertheless, since there is a dual role of NLRP1 activities, namely, inflammasome-mediated IL-1b regulation and the caspase-mediated regulation of apoptosis and pyroptosis, it is very likely that the fine-tuning of these activities, in different cells of lung parenchyma, contributes to protection. 

It is also important to say that our study has several limitations. Our study, as most of the genetic case-control studies on complex diseases, such as COPD, suffers from the heterogeneity of the tested cohorts, and in our case, the presence of different comorbidities and smoking behaviors/statuses. COPD is a smoking related disease, and in our study 91.8% of the COPD patients were current or former smokers. It is very difficult to recruit a comparable control population without respiratory problems or other comorbidities. Also, our control population consisted of healthy blood donors, and included only 25.5% current or former smokers. Finally, men dominated in the COPD population (69.2%), and among blood donors this was even more true (87.3%). Therefore, we took these differences into account in our analyses by adjusting for age, sex, and smoking status. Furthermore, the COPD patients represented many different comorbidities, 54.6% had at least two comorbidities, thus creating many different phenotypes with different level of severity of the disease. Presence of different comorbidities, especially those associated with chronic inflammation, should be taken with special caution. Also, the size of the tested cohorts should be significantly larger because the analyses included small subgroups, decreasing the power to detect associations. Larger studies with well-characterized study populations are needed to fully clarify the associations observed in our study and to investigate the functional basis of the associations. Therefore, it is important to say that our results should be carefully interpreted. Still, we believe that results presented here have contributed, at least in part, to the progress in elucidating the pathogenesis of COPD, in the context of the genetic variability of the receptors of innate immune response, like NLRPs.

## Figures and Tables

**Table 1:** Demographic characteristics of the tested population.

Characteristic	Chronic Obstructive Pulmonary Disease (COPD) Patients	Healthy Controls
Sex		
Male	487 (69.2)	1116 (87.3)
Female	217 (30.8)	163 (12.7)
Age, year, median (SD, minimum and maximum)	61 (8.25; 33–85)	38 (12; 16–66)
Smoking History		
Non-smoker	56 (8.1)	954 (74.4)
Former	424 (61.5)	3 (0.2)
Active	209 (30.3)	325 (25.3)
Missing data	15 (2.1)	1 (0.1)

Data are presented as number (%).

**Table 2:** The clinical characteristics in the cohort of COPD patients and the frequency of the comorbidities.

Characteristic	COPD Patients
Smoking, pack-year, median (range)	40 (0.8–160)
GOLD classification	
A	172 (26.1)
B	171 (25.9)
C	172 (26.1)
D	145 (21.9)
FEV_1_/FVC, %, mean (SD)	56.66 (21.6)
FEV_1_, %, mean (SD)	55.13 (22.0)
Cough	597/704 (84.8)
mMRC ^1^	
0	59 (8.4)
1	168 (23.9)
2	238 (33.9)
3	166 (23.6)
4	72 (10.2)
Annual exacerbation rate	
0	234 (33.2)
2	346 (49.1)
>2	124 (17.6)
Hospital admission	276 (39.2)
Number of comorbidities	
0	117 (16.6)
1	203 (28.8)
2	201 (28.6)
≥3	183 (26)
Comorbidities	
Pulmonary hypertension	49 (7%)
Arterial hypertension	353 (50.1%)
Lung cancer	72 (10.2%)
Cardiovascular diseases	196 (27.8%)
Diabetes mellitus	100 (14.2%)
Extrapulmonary neoplasms	63 (8.9%)
Chronic gastritis /GERD	71 (10.1%)
Chronic kidney disease	29 (4.1%)
Osteoporosis	35 (5%)
Psychiatric disorders	37 (5.3%)

Data are presented as number (%). ^1^ 0, breathless with strenuous exercise; 1, shortness of breath when hurrying on the level or walking up a slight hill; 2, shortness of breath when walking its own pace on the level; 3, shortness of breath after walking about 100 m; 4, breathless when dressing. GOLD, Global Initiative for Chronic Obstructive Lung Disease; FEV_1_, forced expiratory volume in one second; FVC, forced vital capacity; mMRC, modified Medical Research Council dyspnea; GERD, gastroesophageal reflux disease.

**Table 3:** List of the single nucleotide polymorphisms (SNPs) analyzed in the study, minor allele frequency, and the functional consequence according to SIFT and PolyPhen, respectively.

GENE	SNP	MAF	Amino Acid Change	Domain	SIFT	PolyPhen
*NLRP1*	rs12150220_A_T	0.231 (T)	L155H		Deleterious ^1^	possibly damaging ^1^
*NLRP1*	rs35596958_T_C	0.046 (C)	M1119V	FIIND	tolerated	benign
*NLRP2*	rs1043673_C_A	0.416 (A)	A1052E		tolerated	benign
*NLRP3*	rs35829419_C_A	0.022 (A)	Q705K		tolerated	benign
*NLRP4*	rs441827_C_T	0.383 (T)	A144T		tolerated	benign
*NLRP4*	rs12462372_G_A	0.059 (A)	R708H	LRR	tolerated	benign/probably damaging ^2^
*NLRP4*	rs17857373_G_C	0.013 (C)	P390Q	NACHT	tolerated	benign
*NLRP4*	rs302453_A_T	0.219 (T)	Q925L	LRR	tolerated	benign
*NLRP5*	rs10409555_G_A	0.258 (A)	V1181I	LRR	tolerated	benign
*NLRP5*	rs16986899_T_C	0.265 (C)	M912T	LRR	tolerated	benign
*NLRP5*	rs471979_G_C	0.125 (C)	M459I	NACHT	tolerated	benign
*NLRP6*	rs56159585_T_A	0.299 (A)	Y361F	NACHT	tolerated	benign
*NLRP6*	rs77447196_C_G	0.217 (G)	P244A	NACHT	tolerated	benign
*NLRP8*	rs306457_C_G	0.275 (G)	Stop lost		tolerated	benign
*NLRP8*	rs306481_G_A	0.494 (A)	K937R	LRR	tolerated	benign
*NLRP11*	rs12461110_G_A	0.243 (A)	P438L	NACHT	tolerated	benign/possibly damaging/probably damaging ^3^
*NLRP11*	rs299163_A_C	0.047 (C)	A188S	NACHT	tolerated	benign
*NLRP12*	rs34436714_A_C	0.267 (C)	G39V	NACHT	deleterious ^1^	benign
*NLRP12*	rs34971363_G_C	0.051 (C)	F402L	NACHT	deleterious ^1^	probably damaging ^2^
*NLRP13*	rs303997_C_T	0.335 (T)	Q247R	NACHT	deleterious ^1^	benign

^1^ In all transcripts. ^2^ Depending on transcript (probably damaging in one of the protein coding transcripts). ^3^ Depending on transcript (possible damaging in one of the protein coding transcripts; probably damaging in two nonsense mediated decay transcripts). MAF, minor allele frequency in Croatian population; SIFT, Sorting Intolerant from Tolerant; PolyPhen, Polymorphism Phenotyping.

**Table 4:** Genotype frequencies of tested single nucleotide polymorphisms (SNPs) in the COPD cases and the control population and their associations with COPD adjusted for age, gender and smoking status.

		Adjusted for Age/Gender/Smoking			
SNP	Genotype	COPD Cases	Controls	OR (95%CI ^2^) ^1^	*p*
rs12150220	A/A	113 (29.20%)	307 (24.64%)	1.00	
	A/T	190 (49.10%)	642 (51.52%)	0.54 (0.31–0.95)	0.03 *
	T/T	84 (21.71%)	297 (23.84%)	0.59 (0.28–1.26)	0.17
	A/T+T/T	274 (70.80%)	939 (75.36%)	0.55 (0.32–0.95)	0.03 *
rs35596958	T/T	335 (86.79%)	1086 (87.16%)	1.00	
	C/T	49 (12.69%)	160 (12.84%)	0.86 (0.42–1.78)	0.69
	C/C	2 (0.52%)	0 (0.00%)	-	-
rs1043673	C/C	152 (39.79%)	471 (37.83%)	1.00	
	A/C	177 (46.34%)	589 (47.31%)	1.13 (0.65–1.95)	0.67
	A/A	53 (13.87%)	185 (14.86%)	0.92 (0.42–2.01)	0.83
rs35829419	C/C	346 (89.41%)	1141 (91.35%)	1.00	
	A/C	39 (10.08%)	107 (8.57%)	1.07 (0.44–2.58)	0.88
	A/A	2 (0.52%)	1 (0.08%)	-	0.80
rs441827	C/C	155 (40.16%)	464 (37.09%)	1.00	
	T/C	168 (43.52%)	598 (47.80%)	0.82 (0.47–1.44)	0.49
	T/T	63 (16.32%)	189 (15.11%)	1.02 (0.50–2.10)	0.95
rs12462372	G/G	354 (91.00%)	1092 (88.21%)	1.00	
	A/G	34 (8.74%)	143 (11.55%)	0.36 (0.15–0.90)	0.03 *
	A/A	1 (0.26%)	3 (0.24%)	0.29 (0.00–949.77)	0.77
	A/G+A/A	35 (9.00%)	146 (11.79%)	0.36 (0.15–0.90)	0.03 *
rs17857373	G/G	359 (92.76%)	1162 (92.89%)	1.00	
	C/G	26 (6.72%)	88 (7.03%)	0.96 (0.34–2.69)	0.94
	C/C	2 (0.52%)	1 (0.08%)	-	-
rs302453	A/A	215 (55.41%)	681 (54.83%)	1.00	
	A/T	159 (40.98%)	471 (37.92%)	1.59 (0.94–2.71)	0.09
	T/T	14 (3.61%)	90 (7.25%)	0.31 (0.09–0.99)	0.05
rs10409555	G/G	212 (54.78%)	664 (53.12%)	1.00	
	A/G	142 (36.69%)	495 (39.60%)	0.59 (0.34–1.03)	0.06
	A/A	33 (8.53%)	91 (7.28%)	1.75 (0.73–4.20)	0.21
rs16986899	T/T	268 (69.79%)	857 (68.89%)	1.00	
	C/T	104 (27.08%)	348 (27.97%)	1.17 (0.66–2.07)	0.60
	C/C	12 (3.13%)	39 (3.14%)	0.77 (0.19–3.06)	0.71
rs471979	G/G	293 (79.62%)	962 (80.37%)	1.00	
	C/G	68 (18.48%)	219 (18.30%)	0.72 (0.36–1.43)	0.35
	C/C	7 (1.90%)	16 (1.34%)	4.14 (0.93–18.49)	0.06
rs56159585	T/T	320 (82.69%)	969 (79.23%)	1.00	
	A/T	61 (15.76%)	238 (19.46%)	0.54 (0.27–1.07)	0.08
	A/A	6 (1.55%)	16 (1.31%)	0.11 (0.00–4.43)	0.24
rs77447196	C/C	265 (68.48%)	823 (66.42%)	1.00	
	C/G	117 (30.23%)	363 (29.30%)	0.72 (0.40–1.29)	0.27
	G/G	5 (1.29%)	53 (4.28%)	0.38 (0.07–2.12)	0.27
rs306457	C/C	227 (58.81%)	757 (60.71%)	1.00	
	C/G	134 (34.72%)	413 (33.12%)	1.08 (0.63–1.85)	0.77
	G/G	25 (6.48%)	77 (6.17%)	0.39 (0.12–1.30)	0.12
rs306481	G/G	142 (36.50%)	405 (32.58%)	1.00	
	A/G	190 (48.84%)	604 (48.59%)	0.95 (0.55–1.65)	0.86
	A/A	57 (14.65%)	234 (18.83%)	0.60 (0.27–1.33)	0.21
rs12461110	G/G	158 (41.15%)	501 (40.14%)	1.00	
	A/G	160 (41.67%)	568 (45.51%)	0.76 (0.44–1.33)	0.34
	A/A	66 (17.19%)	179 (14.34%)	1.26 (0.61–2.61)	0.54
rs299163	A/A	349 (89.95%)	1066 (85.76%)	1.00	
	A/C	38 (9.79%)	172 (13.84%)	0.93 (0.46–1.90)	0.85
	C/C	1 (0.26%)	5 (0.40%)	0.02 (0.00–11710.48)	0.57
rs34436714	C/C	251 (65.88%)	771 (61.98%)	1.00	
	A/C	117 (30.71%)	407 (32.72%)	0.62 (0.35–1.08)	0.09
	A/A	13 (3.41%)	66 (5.31%)	0.40 (0.11–1.43)	0.16
rs34971363	G/G	325 (83.98%)	1049 (83.79%)	1.00	
	C/G	60 (15.50%)	191 (15.26%)	1.26 (0.62–2.58)	0.52
	C/C	2 (0.52%)	12 (0.96%)	0.41 (0.01–31.83)	0.69
rs303997	C/C	124 (32.12%)	418 (33.63%)	1.00	
	C/T	201 (52.07%)	630 (50.68%)	1.13 (0.65–1.98)	0.67
	T/T	61 (15.80%)	195 (15.69%)	1.09 (0.51–2.30)	0.83

* *p* values <0.05 ^1^ OR, odds ratio ^2^ CI, confidence interval.

## Supplementary Materials

The following are available online at https://www.mdpi.com/2073-4425/10/10/783/s1, Table S1: Distribution of the *NLRP* polymorphisms between different GOLD groups (ABCD). Figure S1: Association of genotypes of NLRP polymorphisms with patients’ overall survival estimated by the Kaplan–Meier method.
