# Supplementary material for: Association of NLRP1 Coding Polymorphism with Lung Function and Serum IL-1β Concentration in Patients Diagnosed with Chronic Obstructive Pulmonary Disease (COPD)
Source: Genes (Basel). 2019 Oct 9;10(10):783. doi: 10.3390/genes10100783 (PMC6826440; doi:10.3390/genes10100783)

Article_Supplementary

Association of the *NLRP1* coding polymorphism with the lung function and serum IL-1β concentration in the patients diagnosed with chronic obstructive pulmonary disease (COPD)

Petar Ozretić[1¶], Miguel Inacio da Silva Filho[2¶], Calogerina Catalano[2], Irena Sokolović[3], Andrea Vukić-Dugac[3], Maja Šutić[1], Matea Kurtović[1], Gordana Bubanović[3], Sanja Popović-Grle[3], Sanda Skrinjarić-Cincar[4,5], Oliver Vugrek[1], Irena Jukić[6], Lada Rumora[7], Martina Bosnar[8] Miroslav Samaržija[3], Robert Bals[9], Marko Jakopović[3], Asta Försti[2,*] and Jelena Knežević[1,*]

[1] Ruđer Bošković Institute, Division of Molecular Medicine, Zagreb, Croatia

[2] Division of Molecular Genetic Epidemiology, DKFZ, Heidelberg, Germany

[3] Department for Respiratory Diseases Jordanovac, University of Zagreb School of Medicine, University Hospital Centre Zagreb, Zagreb, Croatia

[4] J. J. Strossmayer" University School of Medicine, Osijek, Croatia

[5] Universitiy Hospital Center Osijek, Department of Pulmology, Osijek, Croatia

[6] Croatian Institute of Transfusion Medicine, Zagreb, Croatia

[7] Department of Medical Biochemistry and Hematology, Faculty of Pharmacy and Biochemistry, University of Zagreb, Zagreb, Croatia

[8] Fidelta d.o.o., Prilaz baruna Filipovića 29, 10 000 Zagreb, Croatia

[9] Department of Internal Medicine V - Pulmonology, Allergology, Intensive Care Medicine, Saarland University, Homburg, Germany

¶ These authors contributed equally

* These authors share senior authorship

* Correspondence: jknezev@irb.hr, Tel.: ++385 1 4560 964

Received: date; Accepted: date; Published: date

Table S1. Distribution of the *NLRP* polymorphisms between different GOLD groups (ABCD).

SNP	GOLD	Genotype			p[1]
rs12150220		AA	AT	TT	
	A	48 (35.04)	52 (21.49)	29 (24.79)	**0.049**
	B	41 (29.93)	70 (28.93)	41 (35.04)	
	C	24 (17.52)	65 (26.86)	28 (23.93)	
	D	24 (17.52)	55 (22.73)	19 (16.24)	
rs35596958		TT	CT	CC	
	A	111 (25.69)	17 (26.98)	0 (0)	0.91
	B	133 (30.79)	18 (28.57)	1 (50)	
	C	103 (23.84)	14 (22.22)	1 (50)	
	D	85 (19.68)	14 (22.22)	0 (0)	
rs1043673		CC	AC	AA	
	A	48 (26.09)	64 (27.71)	14 (19.18)	0.34
	B	63 (34.24)	67 (29)	22 (30.14)	
	C	41 (22.28)	58 (25.11)	16 (21.92)	
	D	32 (17.39)	42 (18.18)	21 (28.77)	
rs35829419		CC	AC	AA	
	A	121 (27.25)	7 (14.29)	0 (0)	0.15
	B	130 (29.28)	19 (38.78)	2 (100)	
	C	103 (23.20)	14 (28.57)	0 (0)	
	D	90 (20.27)	9 (18.37)	0 (0)	
rs441827		CC	TC	TT	

	A	51 (26.15)	54 (25.23)	22 (26.19)	0.94
	B	59 (30.26)	65 (30.37)	27 (32.14)	
	C	46 (23.59)	55 (25.7)	16 (19.05)	
	D	39 (20)	40 (18.69)	19 (22.62)	
rs12462372		GG	AG	AA	
	A	116 (26.13)	10 (19.23)	0 (0)	0.57
	B	135 (30.41)	19 (36.54)	0 (0)	
	C	104 (23.42)	12 (23.08)	1 (100)	
	D	89 (20.05)	11 (21.15)	0 (0)	
rs17857373		GG	CG	CC	
	A	118 (25.88)	9 (23.08)	1 (50)	0.80
	B	141 (30.92)	12 (30.77)	1 (50)	
	C	105 (23.03)	12 (30.77)	0 (0)	
	D	92 (20.18)	6 (15.38)	0 (0)	
rs302453		AA	AT	TT	
	A	70 (25.64)	50 (24.63)	8 (42.11)	0.32
	B	76 (27.84)	72 (35.47)	4 (21.05)	
	C	68 (24.91)	43 (21.18)	5 (26.32)	
	D	59 (21.61)	38 (18.72)	2 (10.53)	
rs10409555		GG	AG	AA	
	A	67 (25.28)	52 (27.66)	10 (22.73)	0.32
	B	78 (29.43)	63 (33.51)	12 (27.27)	
	C	59 (22.26)	47 (25)	11 (25)	

	D	61 (23.02)	26 (13.83)	11 (25)	
rs16986899		CC	CT	TT	
	A	87 (25.44)	36 (27.07)	5 (26.32)	0.89
	B	106 (30.99)	41 (30.83)	5 (26.32)	
	C	80 (23.39)	32 (24.06)	3 (15.79)	
	D	69 (20.18)	24 (18.05)	6 (31.58)	
rs471979		GG	CG	CC	
	A	91 (24.20)	22 (23.40)	3 (37.50)	0.98
	B	119 (31.65)	31 (32.98)	2 (25)	
	C	92 (24.47)	22 (23.40)	2 (25)	
	D	74 (19.68)	19 (20.21)	1 (12.50)	
rs56159585		TT	AT	AA	
	A	101 (25)	22 (25.88)	3 (42.86)	0.22
	B	131 (32.43)	20 (23.53)	2 (28.57)	
	C	88 (21.78)	28 (32.94)	2 (28.57)	
	D	84 (20.79)	15 (17.65)	0 (0)	
rs77447196		CC	CG	GG	
	A	81 (23.62)	45 (31.03)	1 (14.29)	0.11
	B	111 (32.36)	40 (27.59)	0 (0)	
	C	84 (24.49)	29 (20)	4 (57.14)	
	D	67 (19.53)	31 (21.38)	2 (28.57)	
rs306457		CC	CG	GG	
	A	74 (25.61)	45 (26.47)	9 (26.47)	0.34

	B	81 (28.03)	59 (34.71)	10 (29.41)	
	C	78 (26.99)	34 (20)	5 (14.71)	
	D	56 (19.38)	32 (18.82)	10 (29.41)	
rs306481		GG	AG	AA	
	A	31 (17.61)	80 (32.52)	18 (23.68)	**0.0002**
	B	56 (31.82)	78 (31.71)	20 (26.32)	
	C	49 (27.84)	55 (22.36)	12 (15.79)	
	D	40 (22.73)	33 (13.41)	26 (34.21)	
rs12461110		GG	AG	AA	
	A	51 (24.17)	49 (24.50)	29 (34.52)	0.34
	B	65 (30.81)	69 (34.50)	19 (22.62)	
	C	53 (25.12)	41 (20.50)	21 (25)	
	D	42 (19.91)	41 (20.50)	15 (17.86)	
rs299163		AA	AC	CC	
	A	117 (26.29)	11 (21.57)	0 (0)	0.71
	B	132 (29.66)	20 (39.22)	1 (50)	
	C	106 (23.82)	11 (21.57)	1 (50)	
	D	90 (20.22)	9 (17.65)	0 (0)	
rs34436714		CC	AC	AA	
	A	80 (24.92)	41 (27.52)	5 (26.32)	0.91
	B	104 (32.40)	41 (27.52)	7 (36.84)	
	C	77 (23.99)	36 (24.16)	3 (15.79)	
	D	60 (18.69)	31 (20.81)	4 (21.05)	

rs34971363		GG	CG	CC	
	A	110 (26.25)	18 (23.68)	0 (0)	0.85
	B	128 (30.55)	23 (30.26)	1 (50)	
	C	100 (23.87)	18 (23.68)	0 (0)	
	D	81 (19.33)	17 (22.37)	1 (50)	
rs303997		CC	CT	TT	
	A	43 (26.22)	63 (25)	23 (29.11)	0.83
	B	51 (31.10)	78 (30.95)	24 (30.38)	
	C	33 (20.12)	61 (24.21)	20 (25.32)	
	D	37 (22.56)	50 (19.84)	12 (15.19)	

Data are presented as number (%).

[1] Significant p-values (< 0.05) are in bold.

SNP, single nucleotide polymorphism, GOLD, Global Initiative for Chronic Obstructive Lung Disease.

Figure S1: Association of genotypes of NLRP polymorphisms with patients' overall survival estimated by the Kaplan–Meier method. See Table 1 for more details about analyzed SNPs. For every SNP presented are survival curves with p-value for the log-rank test. Tick marks indicate censored cases.

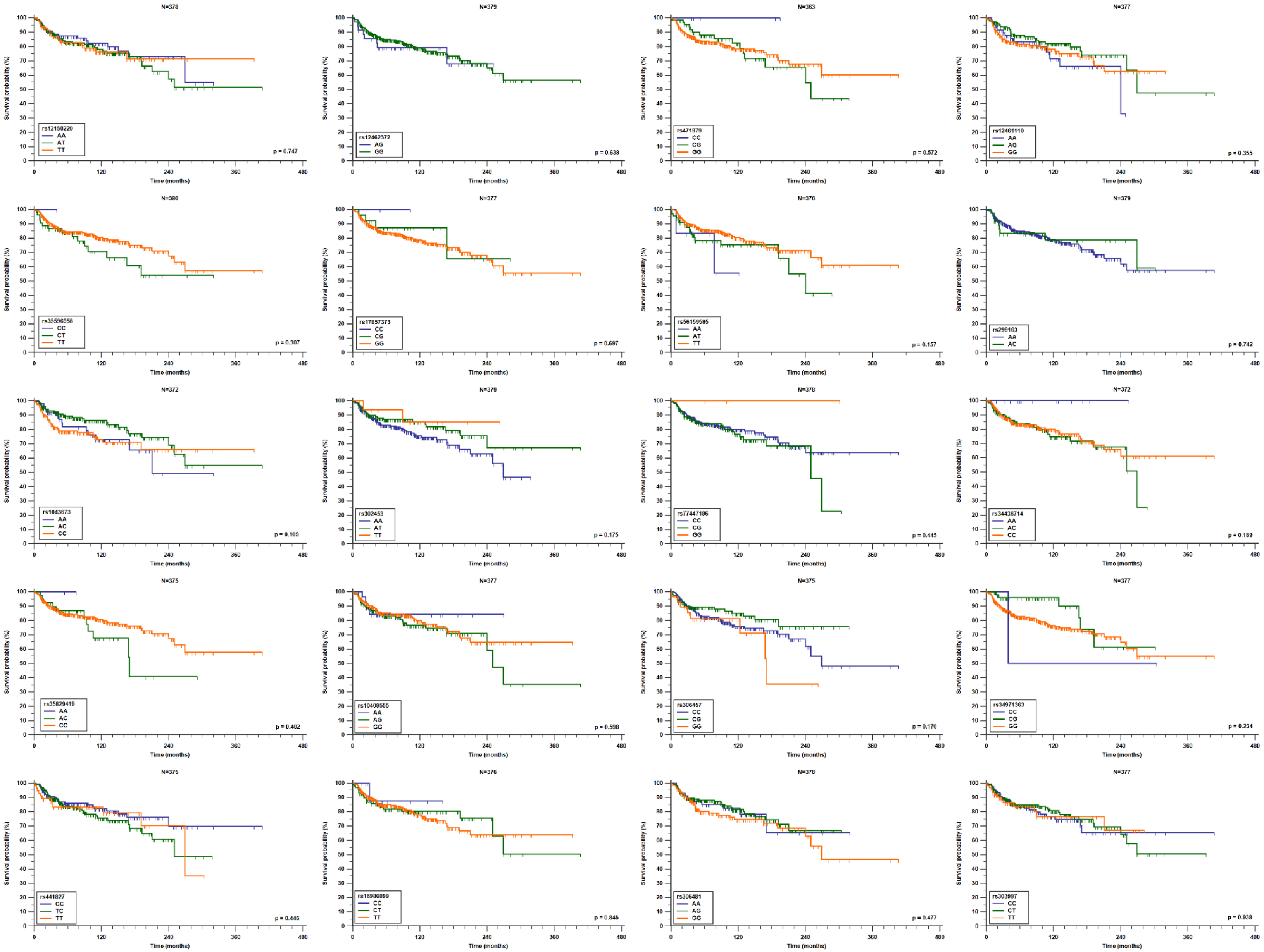

Supplement: Supplementary file 1 [file genes-10-00783-s001.pdf]
